# Audit of Discharge Summary Completion: Identifying Barriers and Implementing Solutions to Improve Timeliness

**DOI:** 10.1192/bjo.2025.10696

**Published:** 2025-06-20

**Authors:** Katie Kopala, Reenie Villeda, Gaurang Surti, Suman Ahmed, Jennifer Luu

**Affiliations:** Leeds and York Partnership Trust, Leeds, United Kingdom

## Abstract

**Aims:** Discharge summaries are an essential part of patient care, ensuring that key medical information, including progress on the ward and treatment plans, is communicated to GPs and community teams. On functional old age psychiatry Wards 3 and 4 at The Mount, Leeds, ensuring timely completion of summaries is important for patient care and safety. The aim is to identify the key factors contributing to delays in writing and sending discharge summaries, providing guidance for implementing changes that support timely completion and help achieve the 7 day target.

**Methods:** We reviewed discharge summaries from January to December 2023 for patients discharged from Wards 3 and 4. The time from discharge to summary completion was recorded and compared against the 7-day target. Summaries were selected based on numerical randomisation, with 11 cases reviewed from Ward 3 and 13 from Ward 4. After data collection, we invited stakeholders to MDTs, where we identified nine key barriers, mapped the current process, clarified development regarding influence and interest, and prioritised two specific changes while exploring potential solutions.

**Results:** The review of discharge summaries from Wards 3 and 4 revealed delays in completion. In Ward 3, none of the 11 reviewed cases had their discharge summaries completed within the 7-day target. In Ward 4, 23% of the 13 reviewed cases met this target. These delays can negatively impact patient care by slowing communication with GPs and community teams. Nine key barriers were identified, and two were prioritised: lack of uninterrupted time and delays in the allocation of a doctor to complete the discharge summary.

**Conclusion:** This audit identified nine key barriers, including a lack of protected time, unclear doctor allocation, and frequent interruptions due to ward acuity. To address these challenges, we propose implementing a dedicated 4-hour weekly slot for junior doctors to complete summaries, assigning a responsible doctor at the time of discharge, and providing a quiet workspace away from the acute ward but onsite to ensure they remain contactable in an emergency. These changes aim to simplify the process, reduce delays, and support both patient care and staff well-being, helping to achieve the new target of 14 days, extended from the previous target of 7 days.

